# Native Coronal Acromioclavicular Joint Orientation Is Associated with Rockwood Grade in Acute Acromioclavicular Joint Dislocations

**DOI:** 10.3390/jcm15187116

**Published:** 2026-09-14

**Authors:** Hakan Batuhan Kaya, Mehmet Talha Aydın, Murat Birinci, Alper Dünki, Savaş Çamur, Mahmut Enes Kayaalp, Serdar Kamil Çepni

**Affiliations:** 1 Department of Orthopedics and Traumatology, Ümraniye Training and Research Hospital, University of Health Sciences, Elmalıkent Mahallesi Adem Yavuz Cad. No:1, Istanbul 34764, Türkiye; mtaydin17@gmail.com (M.T.A.); drmuratbirinci@gmail.com (M.B.); alperdunki@gmail.com (A.D.); savascamur@yahoo.com (S.Ç.); drserdarcepni@gmail.com (S.K.Ç.); 2 Department of Orthopedics and Traumatology, Fatih Sultan Mehmet Research and Training Hospital, University of Health Sciences, Hastane Sokak No:1/9, Istanbul 34752, Türkiye; mek@mek.md

**Keywords:** acromioclavicular joint, Rockwood classification, orientation angle, Zanca radiograph, shoulder injuries

## Abstract

**Background:** The Rockwood classification is the most widely used radiographic framework for grading acromioclavicular (AC) joint dislocations. However, native anatomical variation may be associated with differences in the radiographic appearance of AC joint dislocations. **Purpose:** To evaluate the association between the acromioclavicular joint coronal orientation angle (AJCO) and Rockwood grade and to characterize the relationship between native coronal orientation and radiographic grade. **Methods:** This retrospective observational radiographic study included 198 adults with acute AC joint dislocations classified as Rockwood II, III, or V on standardized bilateral Zanca radiographs. AJCO was measured on the contralateral, uninjured side as the angle between the AC joint orientation line and a vertical reference. Two blinded orthopedic surgeons performed measurements independently, with reliability assessed using intraclass correlation coefficients. AJCO was compared across Rockwood groups, its monotonic association with grade was evaluated using Spearman correlation, and exploratory receiver operating characteristic analysis was performed for the tested binary contrast. **Results:** AJCO differed significantly across Rockwood injury grades, decreasing progressively from type II to type V injuries (median [IQR]: type II, 18.3° [14.6–26.0°]; type III, 15.3° [12.0–21.3°]; type V, 14.0° [8.6–18.0°]; *p* < 0.001). Consistent with this finding, AJCO demonstrated an inverse weak-to-moderate association with Rockwood grade (Spearman ρ = −0.338, 95% CI −0.453 to −0.214; *p* < 0.001). AJCO showed limited ability to discriminate higher-grade injuries (Rockwood ≥ III), with an AUC of 0.694 (95% CI, 0.612–0.777; *p* < 0.001), characterized by high specificity but low sensitivity at the optimal threshold. Furthermore, lower AJCO values remained significantly associated with higher Rockwood grades after adjustment for age and sex (OR = 0.899 per degree increase, 95% CI, 0.864–0.935; *p* < 0.001). **Conclusions:** Lower AJCO values were associated with higher Rockwood grades in this cohort, but the magnitude of association and discriminatory performance were limited. These findings support AJCO as a morphology-related contextual variable associated with Rockwood grade rather than as a stand-alone diagnostic or treatment-guiding measure.

## 1. Introduction

Acromioclavicular joint dislocations are commonly graded with the Rockwood classification, which remains the dominant framework for describing the extent and direction of displacement after injury. Importantly, Rockwood classification is not restricted to superior translation alone: higher-grade injuries also incorporate posterior or inferior displacement, and contemporary reviews increasingly emphasize that clinically relevant AC joint instability has vertical, horizontal, and rotational components. This is particularly relevant for Rockwood type III AC joint dislocations, for which management remains controversial and radiographic interpretation may influence treatment selection [1,2].

At the same time, the current classification framework is imperfect. Previous studies evaluating the Rockwood system have reported variable interobserver and intraobserver reliability, with agreement ranging from fair to substantial depending on the radiographic protocol, observer experience, and use of quantitative measurements [1,3,4,5]. In particular, visual distinction between adjacent grades and assessment of horizontal instability remain challenging [6,7], whereas standardized bilateral imaging and quantitative measurements may improve reproducibility [8]. These limitations have prompted the development of complementary radiographic descriptors and modified classification systems, including the Acromial Center-to-Dorsal Clavicle (AC-DC) and Glenoid Center-to-Posterior Clavicle (GC-PC) measurements, the V angle, dynamic classifications, and the ABC system, each of which attempts to capture dimensions of displacement that are not fully represented by standard coracoclavicular (CC)-based assessment alone [6,9,10,11,12,13,14,15,16]. Together, these studies highlight the importance of considering how three-dimensional anatomy and displacement are represented on two-dimensional imaging when interpreting the radiographic appearance of AC joint injuries.

Native anatomy may therefore matter more than current classification systems acknowledge. Cadaveric, radiographic, CT, and MRI studies have documented substantial variation in AC joint morphology, including flat, oblique, curved, and overhanging configurations, and anatomical variation has been shown to influence how the AC joint is visualized on imaging [17,18]. These observations raise a clinically relevant but under-studied question: can native coronal AC joint orientation influence the visual expression of post-traumatic displacement on standard radiographs and thereby contribute to variability in Rockwood grading?

The purpose of this study was to determine whether an association exists between the acromioclavicular joint coronal orientation angle (AJCO) and Rockwood grade in patients with acute AC joint dislocations. We hypothesized that lower AJCO values would be associated with assignment to higher Rockwood grades and that this pattern would support native coronal morphology as a potential factor influencing radiographic grading rather than as a direct measure of clinical severity.

## 2. Material and Methods

### 2.1. Study Design and Patient Selection

**Figure 1:**
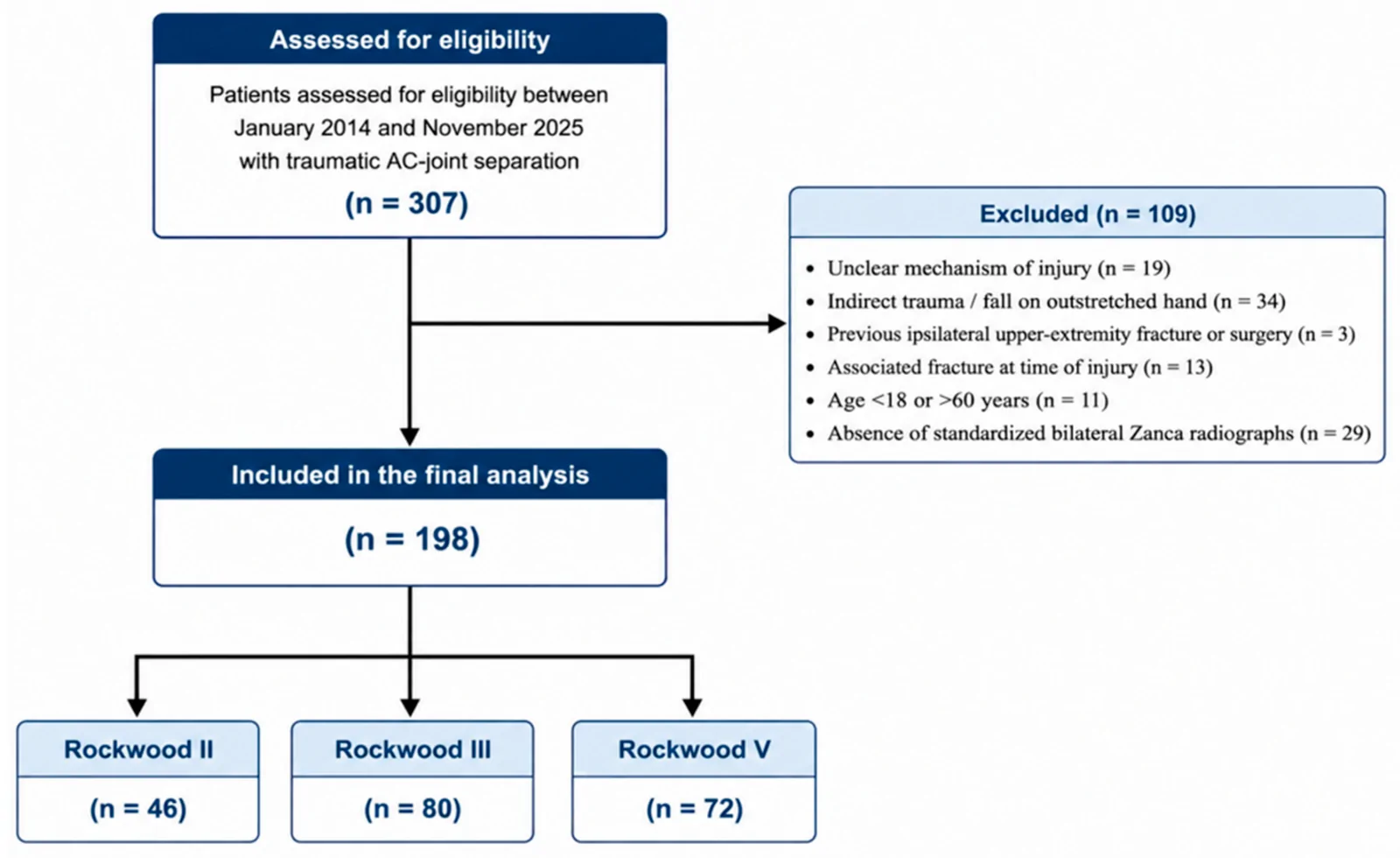
Flow diagram of patient selection.

This retrospective observational radiographic study was approved by the institutional review board and was reported in accordance with the STROBE statement. Consecutive adult patients evaluated at a single tertiary institution between January 2014 and November 2025 for traumatic AC joint dislocation were reviewed. Of 307 screened patients, 198 met inclusion criteria for the final analysis. Eligible patients were 18 to 60 years old, had a documented direct-impact-mechanism injury to the shoulder, and underwent standardized bilateral post-traumatic Zanca radiography at presentation. The analysis was restricted to patients with a documented direct-impact-mechanism injury to the shoulder to reduce heterogeneity related to substantially different injury mechanisms and force vectors. Patients were excluded for unclear or indirect trauma mechanisms, including falls on an outstretched hand, previous ipsilateral upper-extremity fracture or surgery, associated fracture at the time of injury, age outside the predefined range, or absence of standardized bilateral Zanca radiographs (Figure 1).

### 2.2. Radiological Evaluation

Radiographic classification of the injured shoulder was performed by a single orthopedic surgeon using the Rockwood classification system based on standardized bilateral Zanca radiographs. A formal interobserver or intraobserver reliability analysis of Rockwood classification was not performed.

Standardized bilateral Zanca radiographs were obtained with the patient in an upright position and both shoulders positioned symmetrically. The X-ray beam was directed using the institutional Zanca protocol with cephalic angulation. Both AC joints were included to permit side-to-side comparison under the same imaging conditions. Before measurement, radiographs were reviewed for technical adequacy, including visualization of both AC joints and acceptable patient positioning and rotation. Radiographs with substantial asymmetry, rotation, or inadequate visualization of the AC joint that could prevent reliable determination of the AC joint orientation line were excluded from measurement. Because patient positioning and radiographic projection may affect the apparent orientation of the AC joint, the vertical reference used for AJCO was considered an image-based radiographic reference rather than an absolute anatomical vertical axis.

**Figure 2:**
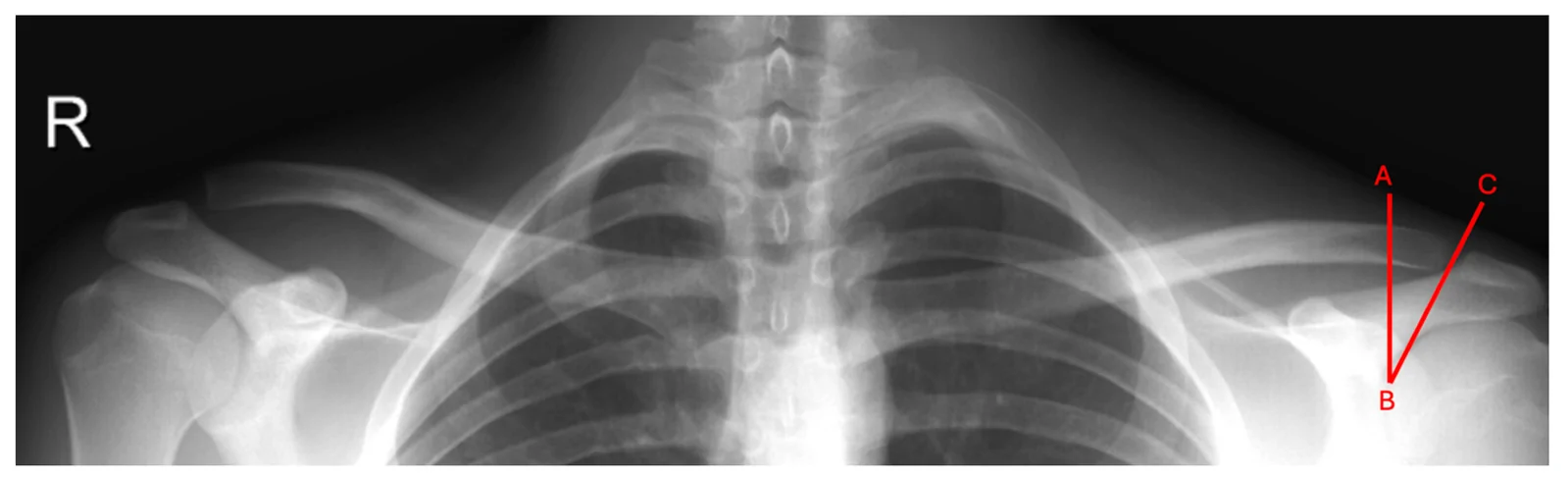
Measurement of the acromioclavicular joint coronal orientation angle (AJCO). The superior and inferior endpoints of the AC joint space were identified and connected to define the AC joint orientation line (B–C). The radiographic vertical reference line (A–B) was constructed on the standardized bilateral Zanca radiograph. AJCO was defined as the acute angle between these two lines. The injured shoulder is displayed separately on the right to distinguish native anatomy from trauma-related displacement. R: Right.

Because the present study aimed to evaluate native morphology rather than trauma-related deformation, AJCO was measured on the contralateral, uninjured shoulder to approximate native coronal AC joint orientation while avoiding trauma-related displacement of the injured side. Measurements were performed using the digital angle measurement tool within the Picture Archiving and Communication System (PACS). To define the coronal orientation of the AC joint, the superior and inferior endpoints of the AC joint space were identified and connected by a straight line (AC joint orientation line). In joints with mildly curved or irregular articular margins, the same superior and inferior endpoints were used, rather than attempting to follow the local contour of the articular surface. AJCO was defined as the acute angle between the AC joint orientation line and the radiographic vertical reference line (Figure 2).

Contralateral comparison is commonly used in the radiographic assessment of acromioclavicular joint injuries, particularly for side-to-side evaluation of acromioclavicular and coracoclavicular relationships [19,20,21]. Accordingly, the uninjured shoulder was considered the best available within-patient surrogate for native anatomy. However, this approach assumes bilateral correspondence of coronal acromioclavicular joint orientation, which has not been specifically established for AJCO.

All AJCO measurements were independently performed by two orthopedic surgeons who were blinded to each other’s measurements. For interobserver reliability, each observer measured AJCO independently across the full cohort. For intraobserver reliability, one observer repeated the measurements after an interval of at least 2 weeks while blinded to prior results.

### 2.3. Statistical Analysis

Statistical analyses were conducted using SPSS software (version 26.0; IBM Corp., Armonk, NY, USA). Continuous variables were assessed for normality using the Shapiro–Wilk test. Normally distributed continuous variables are presented as the mean ± standard deviation, whereas non-normally distributed variables are presented as medians and interquartile ranges (IQRs). Categorical variables are presented as frequencies and percentages.

- Primary Analysis

The primary analysis evaluated the association between the AJCO and Rockwood classification grade. Differences in AJCO values among Rockwood type II, III, and V injuries were assessed using the Kruskal–Wallis test because AJCO was not normally distributed. When significant, pairwise comparisons were performed using Dunn–Bonferroni correction. Effect size for the overall Kruskal–Wallis comparison was reported using epsilon-squared (ε^2^), whereas pairwise nonparametric effect sizes were expressed using Cliff’s delta. The association between AJCO and ordinal Rockwood classification grade was further examined using Spearman rank correlation analysis with 95% confidence intervals estimated using bootstrap resampling. Categorical variables were analyzed using Pearson’s chi-square test.

- Reliability Analysis

Interobserver and intraobserver reliability were assessed using intraclass correlation coefficients (ICCs) based on a two-way random-effects, absolute-agreement model. Corresponding 95% confidence intervals were calculated for all ICC estimates.

- Secondary and Sensitivity Analysis

An exploratory receiver operating characteristic (ROC) analysis was performed to assess the ability of AJCO to discriminate Rockwood II injuries from Rockwood III/V injuries. This dichotomization was selected to contrast the lower-grade Type II pattern with Types III and V, which demonstrate greater coracoclavicular separation and vertical displacement on standard radiographs. The grouping was based on radiographic characteristics and was not intended to represent a treatment-based distinction. The optimal cutoff was identified using the Youden index. This analysis was considered exploratory and was not intended to establish a diagnostic or treatment-guiding threshold.

For the ordinal regression analysis, Rockwood grades II, III, and V were treated as ordered categories because, within the injury patterns represented in the present cohort, these categories correspond to progressively greater vertical displacement and coracoclavicular separation on standard radiographs. This ordinal treatment was restricted to the included Rockwood II, III, and V injuries and should not be interpreted as representing a continuous severity hierarchy across the entire Rockwood classification, particularly because Rockwood IV injuries represent a distinct pattern characterized by posterior displacement and were not included in the cohort.

To evaluate the robustness of the primary findings, an ordinal logistic regression model was fitted under the proportional-odds assumption, with Rockwood grade (II, III, V) as the ordered dependent variable and AJCO, age, and sex as predictors. Because coracoclavicular distance metrics are embedded within the Rockwood grading system, they were not included in the adjusted model to avoid circular adjustment. The proportional-odds assumption was assessed using a Brant-style approximate test comparing coefficients across cumulative cut-points, and results are reported as omnibus and per-variable chi-square statistics. Odds ratios (ORs) with 95% confidence intervals (CIs) and Wald *p* values are reported. Model fit was summarized using Nagelkerke pseudo-R^2^ values and the likelihood-ratio (LR) chi-square test against the intercept-only model.

A sex-stratified sensitivity analysis was additionally performed because sex distribution differed across Rockwood grades.

A two-sided *p* value < 0.05 was considered statistically significant.

## 3. Results

A total of 307 patients were screened and 198 were included in the final cohort. The represented injury spectrum comprised 46 Rockwood II injuries, 80 Rockwood III injuries, and 72 Rockwood V injuries. The overall mean age was 36.2 ± 11.1 years. Age differed significantly across groups (*p* = 0.009), and sex distribution also differed significantly (*p* = 0.011), whereas injury laterality did not differ among grades (*p* = 0.543) (Table 1).

AJCO measurement demonstrated excellent intraobserver reliability (ICC = 0.906, 95% CI: 0.810–0.954; *p* < 0.001) and good interobserver reliability (ICC = 0.898, 95% CI: 0.794–0.950; *p* < 0.001).

**Figure 3:**
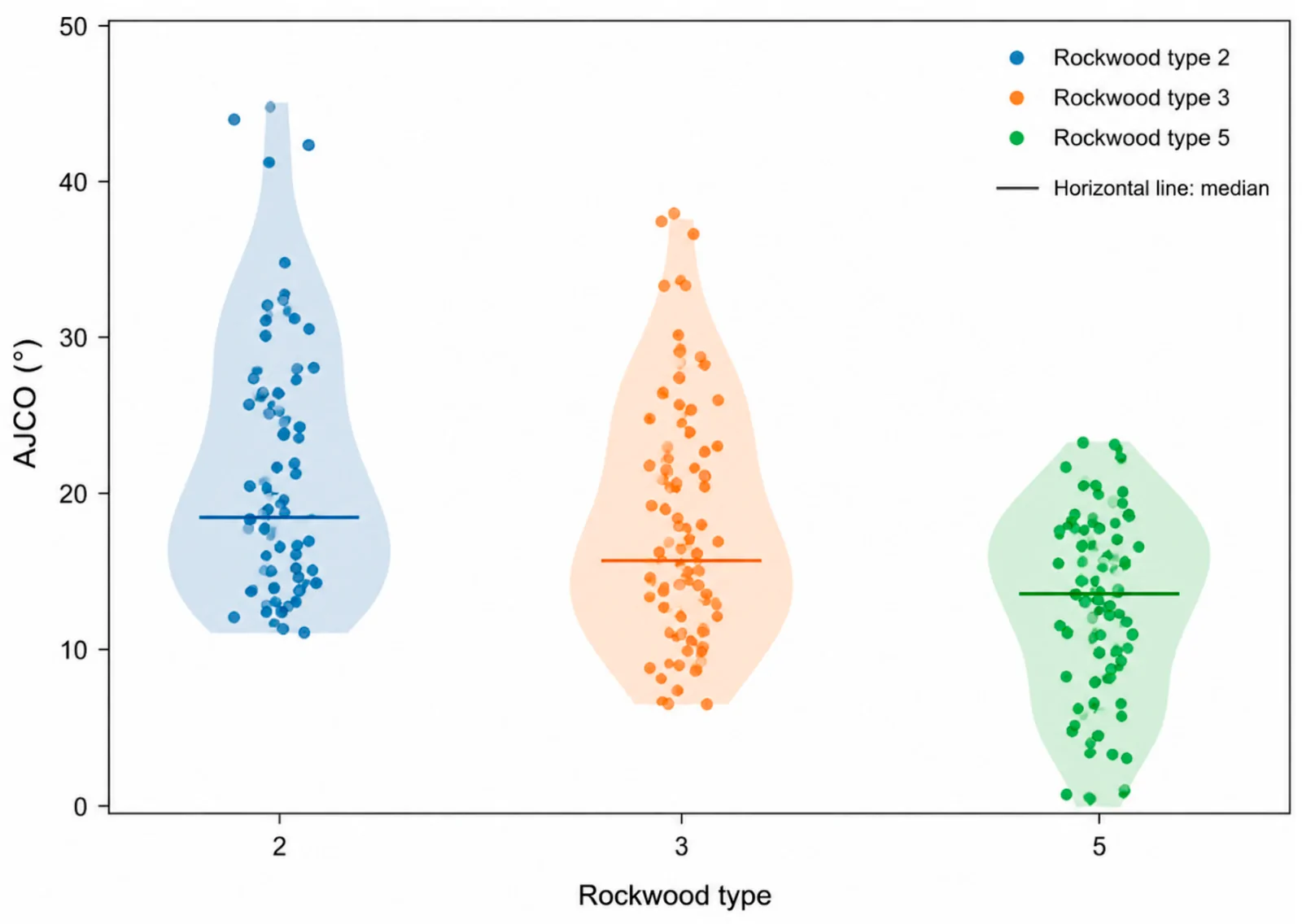
Violin plot with jittered individual data points showing the distribution of AJCO values across Rockwood type II, III, and V injuries. Median values are indicated by horizontal lines. Although AJCO tended to decrease with increasing Rockwood grade, substantial overlap was observed between groups.

AJCO decreased progressively with increasing Rockwood grade. Median AJCO values were 18.3° (IQR, 14.6–26.0°) in Rockwood II injuries, 15.3° (IQR, 12.0–21.3°) in Rockwood III injuries, and 14.0° (IQR, 8.6–18.0°) in Rockwood V injuries (*p* < 0.001) (Table 1). Pairwise Dunn–Bonferroni comparisons demonstrated significant differences between Rockwood II and III (adjusted *p* = 0.019), Rockwood III and V (adjusted *p* = 0.019), and Rockwood II and V injuries (adjusted *p* < 0.001). Effect sizes were small-to-moderate for adjacent grades and moderate for the comparison between Rockwood II and V (Table 2). Although the pattern was monotonic, substantial visual overlap between groups persisted, particularly between Rockwood III and V injuries (Figure 3).

Spearman correlation analysis demonstrated a significant inverse association between AJCO and Rockwood classification grade (ρ = −0.338, 95% CI −0.453 to −0.214; *p* < 0.001). In the exploratory ROC analysis, AJCO demonstrated limited discrimination between Rockwood II and Rockwood III/V injuries (AUC = 0.694, 95% CI 0.612–0.777; *p* < 0.001). The Youden index identified an AJCO value of ≤13.6° as the sample-derived cutoff, with a sensitivity of 40.8% and specificity of 89.1% (Table 3).

In the adjusted ordinal logistic regression model, lower AJCO remained significantly associated with higher Rockwood grade after adjustment for age and sex (OR = 0.899 per degree, 95% CI 0.864–0.935; *p* < 0.001). Age (OR 1.02, 95% CI 1.01–1.04, *p* = 0.010) and female sex (OR 0.25, 95% CI 0.10–0.60, *p* = 0.002) were also significantly associated with Rockwood grade. Model fit was acceptable (Nagelkerke pseudo-R^2^ = 0.24; LR χ^2^(3) = 47.5, *p* < 0.001), and the proportional-odds assumption was not violated (omnibus Brant-style χ^2^(3) = 1.69, *p* = 0.64) (Table 3).

In the sex-stratified sensitivity analysis, lower AJCO remained significantly associated with higher Rockwood grade among male patients (OR = 0.895, 95% CI 0.858–0.933; *p* < 0.001). In female patients, the association was not statistically significant (OR = 0.929, 95% CI 0.815–1.060; *p* = 0.276). However, the female subgroup was small (n = 22), particularly for Rockwood V injuries (n = 5), and these subgroup findings should therefore be interpreted as exploratory (Table 3).

## 4. Discussion

The principal finding of this study is that lower native AJCO values were associated with assignment to higher Rockwood grades among patients with Rockwood II, III, and V AC joint dislocations. Importantly, the magnitude of association was modest rather than strong, and the exploratory discriminative performance of AJCO was limited. The data therefore do not support AJCO as a stand-alone severity classifier. Rather, they support the narrower interpretation that native coronal AC joint morphology is a contextual anatomical variable associated with Rockwood grade.

The current Rockwood classification system remains the dominant framework for radiographic assessment of AC joint injuries and relies substantially on displacement patterns and coracoclavicular relationships on standard radiographs [1]. Nevertheless, increasing evidence suggests that clinically relevant AC joint instability is multidimensional, involving vertical, horizontal, and rotational components [1,12,13,14,15,16]. In response to the recognized limitations of vertical-only assessment, several authors have proposed complementary radiographic descriptors, including the V angle, AC-DC and GC-PC measurements, dynamic instability concepts, and the ABC classification system [12,13,14,15,16]. These approaches emphasize the multidimensional nature of AC joint displacement and the limitations of representing this complexity on two-dimensional radiographs.

The reproducibility of Rockwood grading has varied considerably across previous studies. Some investigations have reported only fair interobserver agreement, whereas others using standardized bilateral radiographs and quantitative measurements have demonstrated substantially better reliability [3,4,5]. More recent studies indicate that reliability is influenced by imaging protocol, measurement strategy, and injury subtype, with particular difficulty in distinguishing certain adjacent categories and characterizing horizontal instability [5,6,9]. These observations highlight that variability in Rockwood grading is likely multifactorial and should not be attributed solely to native AC joint morphology [3,4,5,6,9].

The present findings add to the literature by demonstrating an association between native coronal AC joint morphology and Rockwood grade. Previous studies have documented substantial interindividual variability in AC joint morphology, including flat, oblique, curved, and overhanging configurations [17,18]. Earlier anatomical work by Urist et al. described morphological variation in the AC joint but did not evaluate its association with injury grading [17]. Subsequent studies focused primarily on scapular or clavicular orientation and instability mechanics rather than on native coronal AC joint orientation itself [22,23,24]. To our knowledge, the present study is among the first clinical investigations to specifically examine the relationship between native coronal AC joint orientation and Rockwood grade.

Although lower AJCO values were statistically associated with higher Rockwood grades, substantial overlap remained between groups, particularly between Rockwood III and V injuries. Consistent with this overlap, the exploratory ROC analysis demonstrated only limited discrimination, with relatively high specificity but low sensitivity at the Youden threshold. Therefore, the statistical significance of the observed association should be distinguished from its clinical applicability. The present findings do not support the use of AJCO as an isolated diagnostic, classification, or treatment-guiding parameter in individual patients.

In the adjusted ordinal logistic regression model, AJCO remained significantly associated with higher Rockwood grades after adjustment for age and sex, indicating that the observed association was not fully explained by differences in these variables. Nevertheless, the magnitude of the association remained modest, reinforcing the interpretation that AJCO should be viewed as a contextual morphology-related variable rather than a definitive indicator of injury severity. In the sex-stratified analysis, the association remained significant among men but not among women; however, the small female subgroup substantially limits interpretation of sex-specific effects.

The observed association should not be interpreted as evidence that native AJCO directly determines Rockwood grade. Several alternative explanations remain possible, as lower AJCO may be associated with other anatomical or biomechanical characteristics that were not measured in this study, and the observed relationship may also reflect differences in injury severity, radiographic projection, patient positioning, or other unmeasured factors. Although the association remained significant after adjustment for age and sex, the multivariable model did not account for other potentially relevant anatomical, biomechanical, injury-related, or radiographic factors. Therefore, residual confounding cannot be excluded.

The clinical implications of these findings should be interpreted cautiously. The present data do not support incorporation of AJCO into treatment algorithms or surgical decision-making as an isolated parameter. Its potential clinical relevance requires further evaluation in studies incorporating advanced imaging, dynamic instability assessment, independent anatomical reference standards, and clinical outcomes.

Several limitations of this study should be emphasized. First, the retrospective design precludes causal inference, and residual confounding by unmeasured anatomical, biomechanical, injury-related, or radiographic factors cannot be excluded, as the adjusted analysis included only age and sex. Second, only Rockwood II, III, and V injuries were represented; therefore, the findings cannot be generalized to the full spectrum of AC joint injuries. Third, the cohort was restricted to patients with a documented direct-impact-mechanism injury to the shoulder. Although this criterion was intended to reduce heterogeneity related to different injury mechanisms and force vectors, it may limit the generalizability of the findings to AC joint injuries resulting from indirect mechanisms. Fourth, AJCO was measured on the contralateral, uninjured shoulder as a surrogate for the native morphology of the injured side. Although contralateral comparison is well established in radiographic assessment of AC joint injuries, bilateral symmetry of coronal AC joint orientation has not been specifically validated. Therefore, side-to-side differences in native AJCO cannot be excluded. Fifth, the study relied exclusively on two-dimensional Zanca radiographs without CT or MRI correlation; therefore, three-dimensional osseous morphology and the extent of soft-tissue injury could not be assessed. Sixth, the cohort showed marked male predominance, and sex distribution differed among Rockwood groups, which may limit generalizability. Although an exploratory sex-stratified sensitivity analysis was performed, the small female subgroup (n = 22), particularly the limited number of women with Rockwood V injuries (n = 5), substantially limits the statistical power and interpretation of sex-specific findings. Seventh, Rockwood classification was performed by a single observer, and formal interobserver or intraobserver reliability analysis was not available. Given the previously reported variability in the reproducibility of the Rockwood classification, observer-related variability in assigned Rockwood grade cannot be excluded. Finally, clinical outcomes and treatment responses were not evaluated; consequently, the present findings should be restricted to radiographic interpretation and should not be extrapolated to treatment selection or prognosis.

In conclusion, lower native AJCO values were associated with assignment to higher Rockwood grades in this cohort of acute AC joint dislocations. However, the modest association strength, substantial overlap between groups, and limited discriminative performance indicate that AJCO should not be interpreted as a stand-alone classifier of injury severity. Instead, native coronal AC joint morphology should be regarded as a contextual anatomical variable associated with Rockwood grade. Further prospective studies incorporating independent anatomical and clinical reference standards are required to determine the clinical relevance of this association.

## Figures and Tables

**Table 1:** Demographics and baseline radiographic characteristics by Rockwood grade.

	Rockwood Classification				
	Type II (n: 46)	Type III (n: 80)	Type V (n: 72)	Overall (n: 198)	*p*
Age (years)	33 ± 11.9	36.3 ± 10.9	38.1 ± 10.4	36.2 ± 11.1	**0.009 ^a^**
Gender Female Male	11 (23.9%) 35 (76.1%)	6 (7.5%) 74 (92.5%)	5 (6.9%) 67 (93.1%)	22 (11.1%) 176 (88.9%)	**0.011 ^b^**
Side Right Left	24 (52.2%) 22 (47.8%)	33 (41.3%) 47 (58.7%)	35 (48.6%) 37 (51.4%)	92 (46.5%) 106 (53.5%)	0.543 ^b^
Acromioclavicular Joint Coronal Orientation Angle (AJCO)	18.3 (14.6–26.0)	15.3 (12.0–21.3)	14.0 (8.6–18.0)	15.7 (12.1–21.0)	**<0.001 ^a^**
Affected coracoclavicular (CC) distance	12.8 ± 11.8	15.2 ± 4.8	20.5 ± 4.9	16.5 ± 7.7	**<0.001 ^a^**
Contralateral CC distance	10.9 ± 9.6	9.18 ± 2.4	8.42 ± 2.0	9.3 ± 5.11	**0.004 ^a^**
CC distance ratio (%)	117 ± 12.8	164 ± 22.2	248 ± 51.3	183 ± 62.3	**<0.001 ^a^**

^a^: Kruskal–Wallis test; ^b^: Pearson chi-square test. Bolded *p* values indicate statistical significance.

**Table 2:** Pairwise comparisons of acromioclavicular joint coronal orientation angle (AJCO) among Rockwood II, III, and V injuries.

Comparison	AJCO Difference	Adjusted *p* Value	Effect Size
II vs. III	3.4°	**0.019**	Cliff’s δ = 0.29
III vs. V	3.1°	**0.019**	Cliff’s δ = 0.26
II vs. V	6.7°	**<0.001**	Cliff’s δ = 0.49
Overall (II vs. III vs. V)	—	**<0.001**	ε^2^ = 0.106

Abbreviations: AJCO: acromioclavicular joint coronal orientation angle. Bolded *p* values indicate statistical significance. Overall group comparison was performed using the Kruskal–Wallis test, with epsilon-squared (ε^2^) reported as the overall effect size. Pairwise comparisons were performed using the Dunn–Bonferroni test, with Cliff’s delta (δ) reported as the pairwise effect size.

**Table 3:** Association, discrimination, and sensitivity analyses of AJCO across Rockwood grades.

Analysis	Outcome	Predictor(s)	Estimate	95% CI	*p* Value
Spearman correlation	Rockwood II/III/V	AJCO	ρ = −0.338	−0.453 to −0.214	**<0.001**
Exploratory ROC	Type II vs. III/V	AJCO	AUC = 0.694	0.612 to 0.777	**<0.001**
ROC threshold analysis	Type II vs. III/V	AJCO ≤ 13.6°	Sensitivity = 40.8%; Specificity = 89.1%	N/A	N/A
Adjusted ordinal logistic regression	Rockwood II/III/V	AJCO (per degree)	OR = 0.899	0.864 to 0.935	**<0.001**
		Age (per year)	OR = 1.023	1.005 to 1.042	**0.010**
		Female (vs. male)	OR = 0.248	0.102 to 0.602	**0.002**
Sex-stratified sensitivity analysis—males	Rockwood II/III/V	AJCO (per degree)	OR = 0.895	0.858 to 0.933	**<0.001**
		Age (per year)	OR = 1.020	1.000 to 1.040	**0.045**
Sex-stratified sensitivity analysis—females	Rockwood II/III/V	AJCO (per degree)	OR = 0.929	0.815 to 1.060	0.276
		Age (per year)	OR = 1.041	0.994 to 1.090	0.090

Abbreviations: N/A: not applicable, AJCO: Acromioclavicular joint coronal orientation angle; OR: Odds Ratio; ROC: Receiver Operating Characteristic; AUC: Area Under the Curve; CI: Confidence interval. Bolded *p* values indicate statistical significance.

## Data Availability

The datasets generated and/or analyzed during the current study are available from the corresponding author on reasonable request.
